# Enhancing Protein Expression in HEK-293 Cells by Lowering Culture Temperature

**DOI:** 10.1371/journal.pone.0123562

**Published:** 2015-04-20

**Authors:** Chi-Yen Lin, Zhen Huang, Wei Wen, Andrew Wu, Congzhou Wang, Li Niu

**Affiliations:** Department of Chemistry, and Center for Neuroscience Research, University at Albany, SUNY, Albany, New York, 12222, United States of America; Wuhan Bioengineering Institute, CHINA

## Abstract

Animal cells and cell lines, such as HEK-293 cells, are commonly cultured at 37°C. These cells are often used to express recombinant proteins. Having a higher expression level or a higher protein yield is generally desirable. As we demonstrate in this study, dropping culture temperature to 33°C, but not lower, 24 hours after transient transfection in HEK-293S cells will give rise to ~1.5-fold higher expression of green fluorescent protein (GFP) and α-amino-3-hydroxy-5-methyl-4-isoxazolepropionic acid (AMPA) receptors. By following the time course of the GFP-expressing cells growing at 37°C and 33°C from 24 hours after transfection (including 19 hours recovery at 37°C in the normal growth medium), we found that a mild hypothermia (i.e., 33°C) reduces the growth rate of HEK-293S cells, while increasing cellular productivity of recombinant proteins. As a result, green cells remain undivided in a longer period of time. Not surprisingly, the property of a recombinant protein expressed in the cells grown at 33°C is unaffected, as shown by the use of AMPA receptors. We further demonstrate with the use of PC12 cells that this method may be especially useful when a recombinant protein is difficult to express using a chemical-based, transient transfection method.

## Introduction

Human embryonic kidney 293 (HEK-293) cells [1] are a popular mammalian, heterologous expression system for producing recombinant proteins [2]. These cells can be also used either intact or in lipid fragments to study structure and function relationships and pharmacological properties of the membrane proteins that are expressed in these cells. The benefit of using HEK-293 cells for expressing recombinant proteins includes an efficient transfection of plasmid DNAs, faithful translation and processing of proteins [2]. However, in using these cells for expressing membrane proteins, such as ion channels, a low signal is sometimes observed (e.g., a signal can be current amplitude from electrophysiology or radioactivity from binding experiments). Low signal is generally related to a low copy number of a receptor protein expressed in a cell or precisely on the surface of a cell. Consequently, when either intact cells or cellular membrane fragments that harbor the expressed proteins or receptors have to be used for assays of a membrane protein, the concentration of that membrane protein is further “diluted”. The presence of inhibitors in a measurement further exacerbates the low signal problem and may even prevent a signal from being reliably detected and determined. In addition, the use of HEK-293 cells for a large-scale production of membrane proteins for structural determination is still quite a challenge [3,4,5]. Therefore, it will be useful to find new ways of improving the efficiency of protein expression in HEK-293 cells.

For enhancing expression efficiency, one way is to optimize culture condition. Culture condition (e.g., medium and culture temperature), vector, and host are three major factors that affect the expression of recombinant proteins [6]. Changing culture temperature can be beneficial, because temperature affects cell growth, viability, protein synthesis and metabolism. In this context, lowering temperature from 37°C is known to slow cell growth rate [7,8]. However, early studies showed that lowering temperature had no effect [9,10,11] or even a negative effect on protein expression [12,13,14]. In contrast, some later studies using Chinese hamster ovary (CHO) cells have demonstrated that lowering culture temperature to 30–32°C resulted in higher expression of a variety of recombinant proteins [15,16,17,18,19,20]. These studies suggest that the effect of low temperature on protein expression is cell-line specific, and the enhancement of protein expression is mainly linked to the cold-induced growth arrest within the S or G1 phase of the cell cycle [17,18,21,22]. However, more recent work [23,24] suggests that growth arrest under mild hypothermia conditions (30–35°C) and the associated higher yield of protein expression in mammalian cells may be two independent responses. It should be noted, however, almost of all previous studies of the effect of temperature on production of recombinant proteins have been conducted with CHO cells in suspension culture [6,25]. Very few studies have been attempted with HEK-293 cells, though they are widely used in studies of membrane proteins, especially in static cultures for electrophysiological studies of ion channel proteins.

In the present study, we asked whether lowering culture temperature could enhance protein expression yield in HEK-293 cells (specifically the HEK-293S cell line) and if so, how low temperature can be lowered. We chose two proteins in our study: green fluorescent protein (GFP) [26] and the α-amino-3-hydroxy-5-methyl-4-isoxazolepropionic acid (AMPA) receptors [27,28]. By monitoring green cells with transiently expressed GFP, we followed the time course and the fluorescence intensity of GFP expression in cultures that were subject to mild hypothermia conditions as compared with cultures at 37°C. On the other hand, AMPA receptors are one of the three subtypes of the glutamate ion channel receptor family, and mediate fast synaptic neurotransmission in the central nervous system [29]. Using whole-cell recording, we measured the channel activities of AMPA receptors in the absence and presence of an inhibitor with cells growing under a mild hypothermic condition as compared with those growing at 37°C. In addition, we have tested several, commonly used transfection methods for delivery of plasmid DNAs to explore the temperature effect on the same cell line, including calcium phosphate [30] and Lipofectamine 2000. Based on these results, we describe a protocol in which at 33°C, but not lower, the expression of both GFP and AMPA receptors in HEK-293S cells is increased by ~1.5-fold.

## Materials and Methods

### Plasmid DNAs

The cDNA plasmid that contained the GluA2Q_flip_ gene sequence (unedited at the Q/R site, and flip isoform) was used. Another cDNA construct that contained the GluA3_flip_ gene sequence was also used in this study. Both DNA plasmids also contained the SV40 replication origin (8.6 kb) [31,32]. A GFP construct [33] was expressed either alone or together with an AMPA receptor; in the latter case, GFP was co-expressed as a marker for cell selection for whole-cell recording. To enhance AMPA receptor expression, we also co-expressed a plasmid DNA encoding large T-antigen (TAg) [34]. We previously reported the use of simian virus (SV) 40 TAg to enhance receptor expression in HEK-293 cells [33]. All the plasmids were propagated in an *E*. *coli* host (DH5α) and purified using a kit from QIAGEN (Valencia, CA).

### Cell line, cell culture and transient transfection

The HEK-293S cell line was used in all studies [33,35]. However, the HEK-293S cells were grown in static culture because we were interested in expressing GFP for cell count and intensity measurement as well as channel proteins for electrophysiological measurements. As we reported previously, in static culture, the majority of 293S cells were indeed attached to Petri dishes, albeit more loosely than regular 293 or 293T cells [33]. The cells were maintained in Dulbecco's modified Eagle's medium (DMEM, Lonza BioWhittaker, Cat. No. 12-604F, Walkersville, MD) supplemented with 10% fetal bovine serum (FBS, Invitrogen, Cat. No. 10082–147), and 100 U/ml penicillin and 100 μg/ml streptomycin (CORNING Cellgro, Cat. No. 30-002-CI, Manassas, VA) in a 6% CO_2_, humidified incubator. Two incubators (Forma Series II water-jacked) were used simultaneously. The temperature of one incubator was set at 37°C, whereas the other was set at 33°C or 30°C (see below). A thermometer was placed inside an incubator to provide independent readout of the culture temperature.

To examine the effect of a mild hypothermic culture condition on protein expression, we followed a biphasic temperature culture protocol. The HEK-293 cells were maintained at 37°C normally. After each passage, we continued to grow cells at this temperature to obtain a reasonably high cell density for transfection (see details for transfection below). After transfection, Petri dishes were brought back to 37°C for a minimal five hours before they were transferred to a different incubator with lower culture temperature for a period of time before assays (see Results).

In all of the experiments, transient transfection was performed. A number of transfection reagents were used: calcium phosphate [30], Lipofectamine 2000 (Invitrogen, Cat. No. 18324–111, Carlsbad, CA), Lipofectamine LTX & PLUS (Invitrogen, Cat. No. 15338030) and Metafectene EASY (Biontex Laboratories GmbH, Cat. No. T090-10, Munich, Germany). We also used Opti-MEM I Reduced Serum Medium (Invitrogen, Cat. No. 31985), and the complete medium, which referred to DMEM supplemented with 10% FBS and penicillin/streptomycin. For the PC12 cell line (ATCC, Cat. No. CRL-1721, Manassas, VA), we used the RPMI-1640 Medium (ATCC, Cat. No. 30–2001), supplemented with 10% horse serum (Invitrogen, Cat. No. 26050–088) and 5% FBS with penicillin/streptomycin. The Petri dishes were coated with poly-D-lysine hydrobromide (Sigma-Aldrich, Cat. No. Sigma-P6407, St. Louis, MO).

### Whole-cell current recording

Whole-cell recording [36] was used to assess the activity of AMPA receptor channels expressed in HEK-293S cells under mild hypothermic conditions as compared with those grown at 37°C. In this study, we tested two AMPA receptors, i.e., GluA2Q_flip_ and GluA3_flip_; both can form homomeric receptor channels when expressed individually in HEK-293 cells [31,32]. We also measured the ratio of the whole-cell current amplitude in the absence and presence of an inhibitor with the receptor expressed in a lower temperature, and compared that with the one obtained at 37°C.

The procedure for recording AMPA receptor channel activity was previously described [31,32]. In brief, the recording electrodes were pulled from glass capillaries (World Precision Instruments, Sarasota, FL). The electrode resistance was ~3 MΩ when filled with the electrode solution or internal buffer; the electrode solution contained (in mM) 110 CsF, 30 CsCl, 4 NaCl, 0.5 CaCl_2_, 5 EGTA, and 10 HEPES (pH 7.4 adjusted by CsOH). The external bath solution contained (in mM) 150 NaCl, 3 KCl, 1 CaCl_2_, 1 MgCl_2_, 10 HEPES (pH 7.4 adjusted by HCl). Whole-cell recordings were at—60 mV, and 22°C. Specifically, glutamate was applied from a solution flow device to the cell [31,32,37], and the glutamate-induced whole-cell current was recorded using an Axopatch-200B amplifier at cutoff frequency of 2 kHz by a built-in, 4-pole low-pass Bessel filter, and digitized at 5 kHz sampling frequency using a Digidata 1322A from Axon Instruments (Union City, CA). The data were acquired using pCLAMP 9 (also from Axon). Each data point was an average of at least three measurements collected from at least three cells unless otherwise noted. OriginPro 7 (Origin Lab, Northampton, MA) was used for data plotting. Uncertainties refer to standard deviation of the fits unless noted otherwise.

### Fluorescence imaging and quantification

The green fluorescence due to expression of GFP in HEK-293S cells was imaged on a Carl Zeiss Axiovert S200 microscope and captured using a Sony NEX 3 digital camera and an adapter (Model NY1S-EA, MeCan Imaging Inc., Saitama, Japan). The adapter has 1.74× magnification.

Two sizes of cell images were collected in this study. For each 35 mm Petri dish used to express GFP, we took three bright-field images from three randomly chosen locations under a 20× objective lens (therefore, the total magnification was 20 × 1.74 = 34.8×). For the same viewing field, we also took three fluorescence images. On average, a fluorescence image contained several hundred green cells. These 34.8× images were used to count the total green cells from the fluorescence images and the total cells from the bright-field images for calculating the percentage of green cells or the transfection efficiency. Each fluorescence image contained 2,288 × 1,520 pixels, and each pixel was scored to a value possibly ranging from 0 to 255 on 8-bit digital scale using Image J (version 1.46r from http://rsb.info.nih.gov/ij/). The maximum background intensity was 29 on this scale; below it, no green color could be visually recognized. In this case, we assumed that such a cell did not express GFP, at least not appreciably. On the other hand, a typical green cell contained about 3,000 ± 800 pixels, and the fluorescence intensity per pixel was more than 29 but smaller than 255 (i.e., 255 was the maximum intensity value). We found empirically that the fluorescence intensity of any green cell was roughly even. In other words, all of ~3000 pixels used to digitize the fluorescence intensity of a single green cell in fact had similar intensity scale. As an approximation, therefore, we used a single fluorescence scale to reflect the fluorescence intensity of that single green cell. Furthermore, a green cell could possibly be assigned to one of the three fluorescence intensity scales.

We also took three bright-field/fluorescence image sets from three randomly chosen locations under a 5× objective lens (therefore, the total magnification was 5 × 1.74 = 8.7×). These images on average contained thousands of cells and a typical green cell contained ~600 pixels. These images were used to calculate the overall green intensity, an indication for overall protein expression. It was assumed that the fluorescence intensity in a single GFP-expressing cell was linearly proportional to the amount of GFP that cell expressed.

## Results

### HEK-293S cell growth at 37°C, 33°C and 30°C

**Fig 1:**
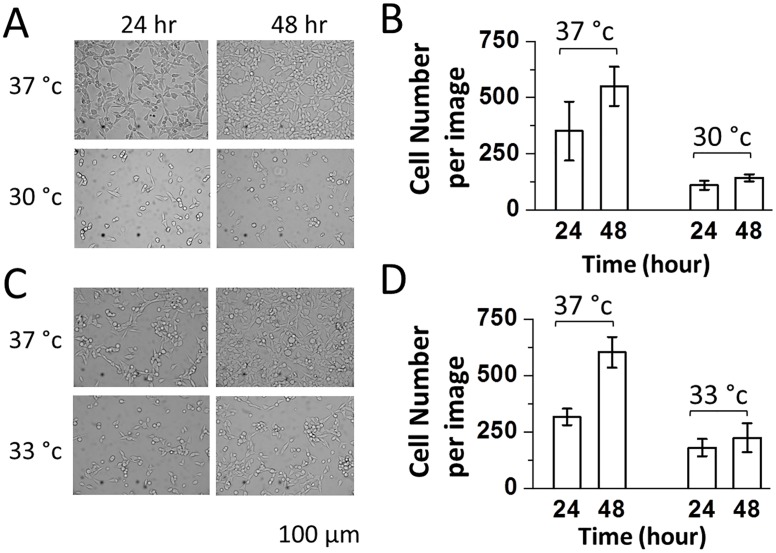
Bright-field images (34.8x magnification) of HEK-293S cells growing at 37°C (upper A and C), 30°C (lower A), and 33°C (lower C). HEK-293S cells were seeded at a density of 5.5 x 10^5^ cells/35 mm Petri dish. The images were taken at 24 and 48 hours using a digital camera mounted on a Carl Zeiss Axiovert S200 microscope (see Methods). Cell number counts per image or 0.22 mm^2^ were plotted in (B) and (D) based on the images shown in (A) and (C), respectively. For each dish, 3 viewing areas were randomly chosen and their images were taken.

To explore whether a mild hypothermic condition would elevate protein expression in HEK-293S cells, we began by expressing and using GFP to establish several parameters that were involved in transient expression. The first experiment was to seed three sets of 35 mm culture dishes with HEK-239S cells at 37°C, 33°C and 30°C. All of the dishes had the same seeding density (~5.5 x10^5^ cells/dish). We observed the following phenomena. First, HEK-293S cells grew significantly slower when incubated at either 30°C (Fig 1A and 1B) or 33°C (Fig 1C and 1D). In contrast, the cells maintained at 37°C grew faster, as evidenced by the increase of cell density of >50% at 48 hours (Fig 1). The cell growth was even slower at 30°C as compared with 33°C. Second, immediately after either passage or transfection, incubating HEK-293S cells at a lower temperature led to a significantly poor cell attachment or a significant cell loss. For example, at 30°C, we observed >60% of cell loss as compared with the 37°C growth temperature. From these experiments, we concluded that 33°C was a reasonable starting point to explore enhancing protein expression under a growth arrest condition. Choosing 30°C would seriously reduce biomass (in addition, lowering culture temperature to 30°C actually had a detrimental effect on protein expression, which will be discussed in detail in “Time Course of GFP Expression).

### Timing of shifting culture temperature from 37°C to 33°C after transient transfection

**Fig 2:**
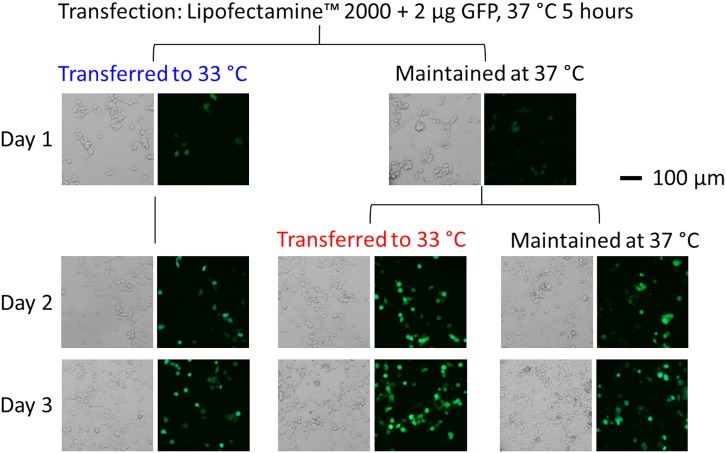
Bright-field and fluorescence images (34.8x magnification) of GFP-expressing HEK-293S cells. The cells were transfected with the GFP plasmid using Lipofectamine 2000. Five hours after transfection (the cells were maintained in a 37°C incubator during this 5-hour period), the medium was replaced. Three sets of dishes were subject to three different ways of maintaining culture temperature, as shown. One set of dishes was transferred to a 33°C incubator. Another set was returned to the 37°C incubator; 19 hours later or 24 hours after transfection, this set of dishes was brought to the 33°C incubator and remained there. The control dishes were maintained at 37°C throughout the experiment. The images were taken from the first day (24 hours after transfection) to the third day.

Normally, HEK-293S cells were maintained at 37°C. After transient transfection with GFP (using Lipofectamine 2000 as an example), we immediately returned the cells to 37°C. Five hours later, we replaced the transfection solution with a normal growth medium in order to minimize cell toxicity. Medium replacement was especially required for calcium-phosphate precipitation [30]. After the medium replacement, we carried out two experiments. In one experiment, we immediately transferred a set of dishes to 33°C (or five hours after transfection); in the other experiment, we returned another set of dishes to 37°C for the cells to recover from transfection. At the 19^th^ hour (or 24 hours after the transfection), we then transferred the second set of dishes from 37°C to 33°C. In addition, we had one set of control dishes maintained at 37°C throughout the experiment. Our results (Fig 2) showed that the shift of the culture temperature from 37°C to 33°C immediately after the medium replacement resulted in an immediate cell growth arrest or a much lower cell density as compared with the control culture at 37°C. In contrast, a 19^th^ hour delay of shifting the culture temperature to 33°C or an overnight recovery at 37°C from transfection prior to lowering temperature to 33°C led to an increase of green fluorescent intensity in the green cells (a quantitative measure will be presented in the next section below). In other words, a delayed temperature shift from 37°C to 33°C by a day resulted in a higher level of GFP expression, as compared with the 37°C control. In contrast, shifting culture to 33°C without overnight delay resulted in lower green cell counts and lower GFP expression (images are shown in the left column of Fig 2). We arrived at this conclusion based on the assumption that GFP fluorescence intensity in HEK-293S cells was linearly correlated to the amount of GFP expressed in those cells.

### Comparison of different methods and the amount of the GFP plasmid used for transfection

**Fig 3:**
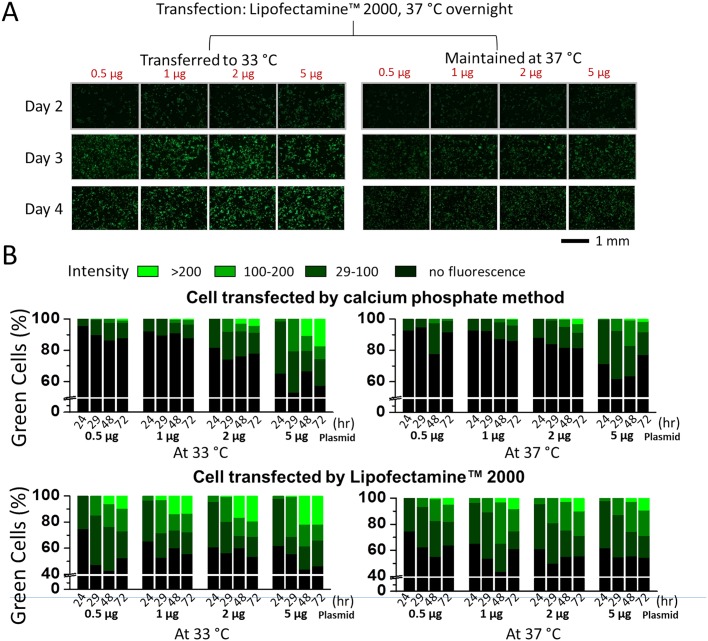
Comparison of different transfection methods and the amount of the GFP plasmid used for transfection. (A) Enhancement of GFP expression in HEK-293S cells that grew at 33°C (on the left), as compared with 37°C (on the right). HEK-293S cells were transfected with varying amounts of the GFP plasmid, as labeled, using Lipofectamine 2000 or by the calcium phosphate method (photos not shown). A set of dishes (on the left) were transferred to a 33°C incubator, 24 hours after transfection and were maintained at 33°C. Shown are green fluorescence images from day 2 to day 4 (8.7x magnification, 1/5 second exposure time). (B) Percentage of the green cells from each of the dishes with varying amounts of the GFP plasmid and the fluorescence intensity from each of three intensity scales. The upper and lower panels are images from cells transfected using calcium phosphate and Lipofectamine 2000, respectively. The images on the right are controls (at 37°C) whereas the images on the left are cells maintained at 33°C (after overnight culture at 37°C). For determining the percentage of green cells, bright-field images for the same dish were also taken (images not shown). In both cases, 34.8x magnification images were used for data analysis. The white colored line represents the line-break. The green cells were further categorized into three levels of brightness. A typical HEK-293S cell in 34.8x magnification contained 3,000 ± 800 pixels. Based on the green fluorescence image, the pixels from the darkest green cell have an intensity of ≥29. Note the green intensity for each pixel in a JPEG file was recorded in an 8-bit byte, which was converted to a scale from 0 to 255. A cell with the average intensity under 29 indicated no GFP fluorescence, at least not appreciably, which we assigned as black color. A cell with an average brightness between 29 and 100 was labeled low green intensity, colored as dark green bar. A cell with an average brightness between 100 and 200 was considered medium intensity, colored as green. Bright green was the color used to label any cell with an average brightness between 200 and 255. The percentage of each green group was calculated based on the total cell count, and the total number of cells was determined from the bright-field view of the same viewing area (images not shown).

Next, we tested different transfection reagents and methods, and varied the amount of the GFP plasmid in an attempt to find the optimal condition of lowering culture temperature to enhance GFP expression. We first chose the Lipofectamine 2000 reagent and calcium phosphate in HEPES-buffered saline solution [38]. Lipofectamine 2000 is a popular transfection reagent (cationic lipid formulation), whereas the calcium phosphate method is the most inexpensive means to deliver gene to cells and is perhaps the benchmark for evaluating transfection efficiency of chemical-based transfection methods [30]. Using the 19-hour delayed shift of temperature from 37°C to 33°C, we carried out two sets of transfections of GFP, i.e., one with Lipofectamine 2000 and the other with calcium phosphate, with respect to its own control at 37°C. In each set of the experiments, the amount of the GFP plasmid for transfection was varied. Shown in Fig 3A are representative green fluorescence images from different cultures with varying plasmid amounts, whereas in Fig 3B, the results of these two sets of experiments were quantified using these images with respect to individual controls.

Four conclusions could be drawn from these data. First, when we compared the data horizontally in Fig 3B (or the same transfection method), we found that regardless of the amount of the plasmid we used, shifting the culture from 37°C to 33°C 24 hours after transfection resulted in higher GFP expression. Second, at 5 μg GFP plasmid culture, transfection efficiency seemed to reach the maximum (the transfection efficiency can be read out from the Y-axis in any of the panels in Fig 3B by either from the bottom of the darker green bar or 100%—the percentage value of the top of the black column: the black column represents the percentage of non-transfected cells). At this plasmid amount, the transfection efficiency between the two methods did not differ significantly. Specifically, with Lipofectamine 2000 the transfection efficiency reached ~60%, whereas with the calcium phosphate method the transfection efficiency was ~50%. In contrast, when we used a lower plasmid amount, we found a major difference in transfection efficiency. For example, in the 1 μg plasmid culture, the transfection efficiency was ~50% with the use of Lipofectamine 2000, as compared to <20% with the use of calcium phosphate method. We also observed a similar result even from 37°C control cultures (see the two corresponding columns on the two vertical panels in Fig 3B). Third, the exposure to and maintenance of the culture under the mild hypothermic condition appeared to alter only green fluorescence intensity without changing the percentage of the total fluorescence (Fig 3A and 3B), at least not appreciably. In other words, shifting the culture to 33°C did not enhance the transfection efficiency but did turn green cells greener—this is evident, for instance, from a taller, the bright green bar (with >200 intensity scale) from the 33°C experiment as compared with the same experiment at the 37°C control. This conclusion was true from the transfection experiment regardless of transfection reagent and the amount of the plasmid used, as well as the time in the culture (after transfection). Fourth, by using the average fluorescence intensity per pixel, we found that the fluorescence intensity increased by 1.2-fold (0.5 μg GFP experiment) and 1.6-fold (5 μg GFP experiment) from cells that were maintained at 33°C as compared with those that grew at 37°C, respectively. These values were calculated from the images taken at the 72 hour after transfection using calcium phosphate (Fig 3B, the 72 hr bars in the two upper panels). For the Lipofectamine 2000 method (the two lower panels at the 72 hr in Fig 3B), the fluorescence intensity increased by 1.5-fold (0.5 μg GFP experiment) and 1.2-fold (5 μg GFP experiment) from 33°C to 37°C, respectively. On average, therefore, GFP expression level was ~1.4-fold higher from cells that were subject to the mild hypothermic treatment.

**Fig 4:**
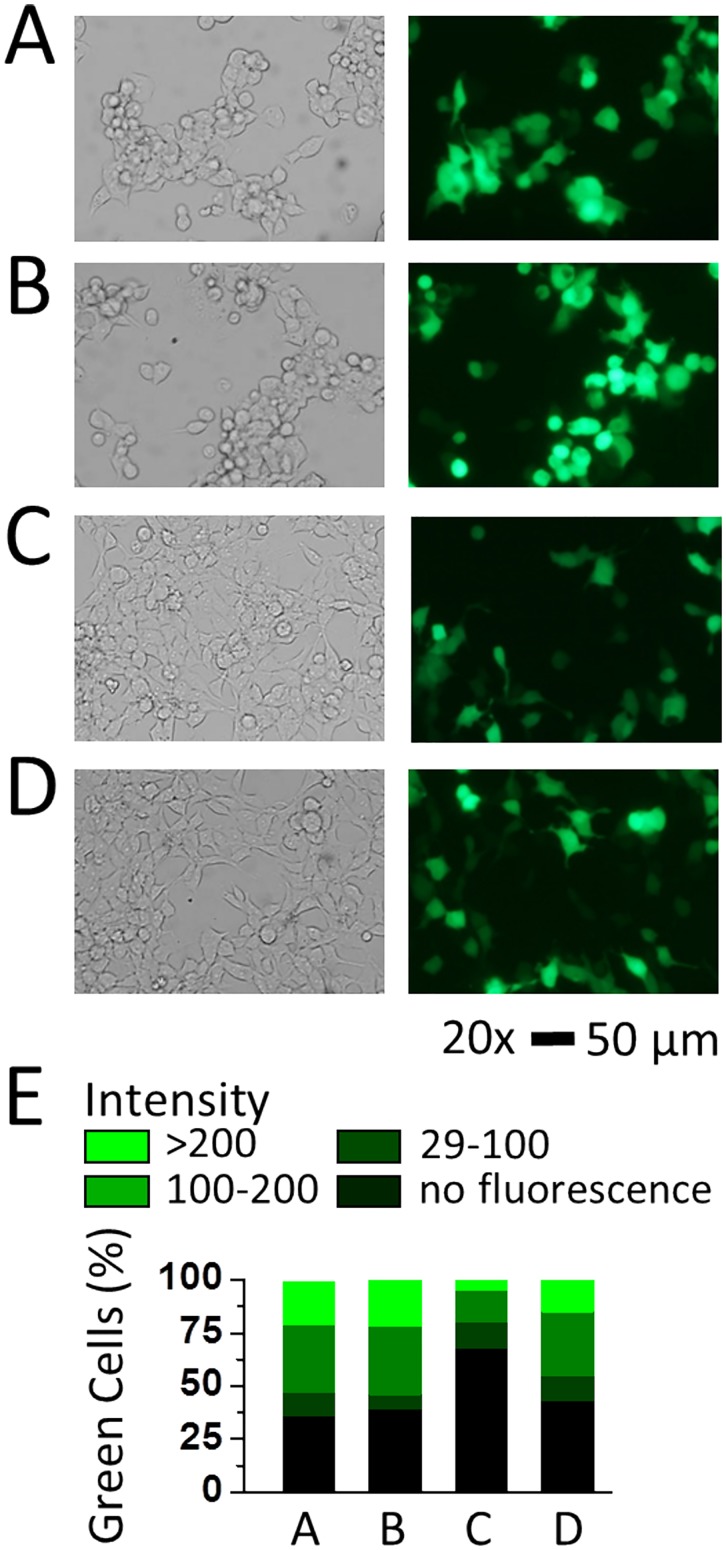
Transfection of the GFP plasmid in HEK-293S cells with different reagents and the expression of GFP at 33°C. Bright-field and fluorescence images (34.8x magnification, scale bar = 50 μm) of HEK-293S cells taken at the 48th hour after transfection. Transfection of HEK-293S cells as in images (A) to (D) was carried out using Lipofectamine 2000, Lipofectamine LTX & PLUS, Metafectene EASY with Opti-MEM and Metafectene EASY with DMEM with 10% FBS, respectively. In each of the transfections, 2 μg GFP plasmid for a 35 mm Petri dish was used. On day 1, all transfected dishes were incubated at 37°C; on day 2, these dishes were transferred to a 33°C incubator. Based on these images, the transfection efficiency for transient expression of GFP was determined to be ~64%, ~61%, ~32% and ~57% from (A) to (D), respectively. (E) The green cells were categorized into high (intensity > 200), middle (intensity 100–200), and low (intensity 29–100) three groups. The black color indicates cells visible in the bright view but can’t been observed under UV (intensity < 29). The percentage of each group was plotted in stacked columns.

Using the same protocol (i.e., 19 hour after the medium replacement or 24 hour after transfection, lowering the culture temperature from 37°C to 33°C), we also tested additional transfection reagents. Shown in Fig 4 are three more experiments that included Lipofectamine LTX Plus, Metafectene EASY with opti-MEM medium and separately with complete medium. We found that the transfection efficiency for GFP in HEK-293S cells was ~61% for Lipofectamine LTX Plus, ~32% for Metafectene EASY with opti-MEM medium and ~57% for Metafectene EASY with complete medium. As the control for this set of experiment, Lipofectamine 2000 reached over 60% transfection efficiency. Although Lipofectamine LTX Plus and Metafectene EASY were supposed to provide better gene expression in commonly used cell lines, our data indicated that Lipofectaime 2000 was perhaps the preferred reagent for the use of HEK-293S cell line. It should be noted that Metafectene EASY did not work well in the Opti-MEM medium or a reduced serum medium in HEK-293S cells, whereas the same reagent with the complete medium almost doubled the transfection efficiency (Fig 4E).

### Time course of GFP expression under the mild hypothermic culture condition

**Fig 5:**
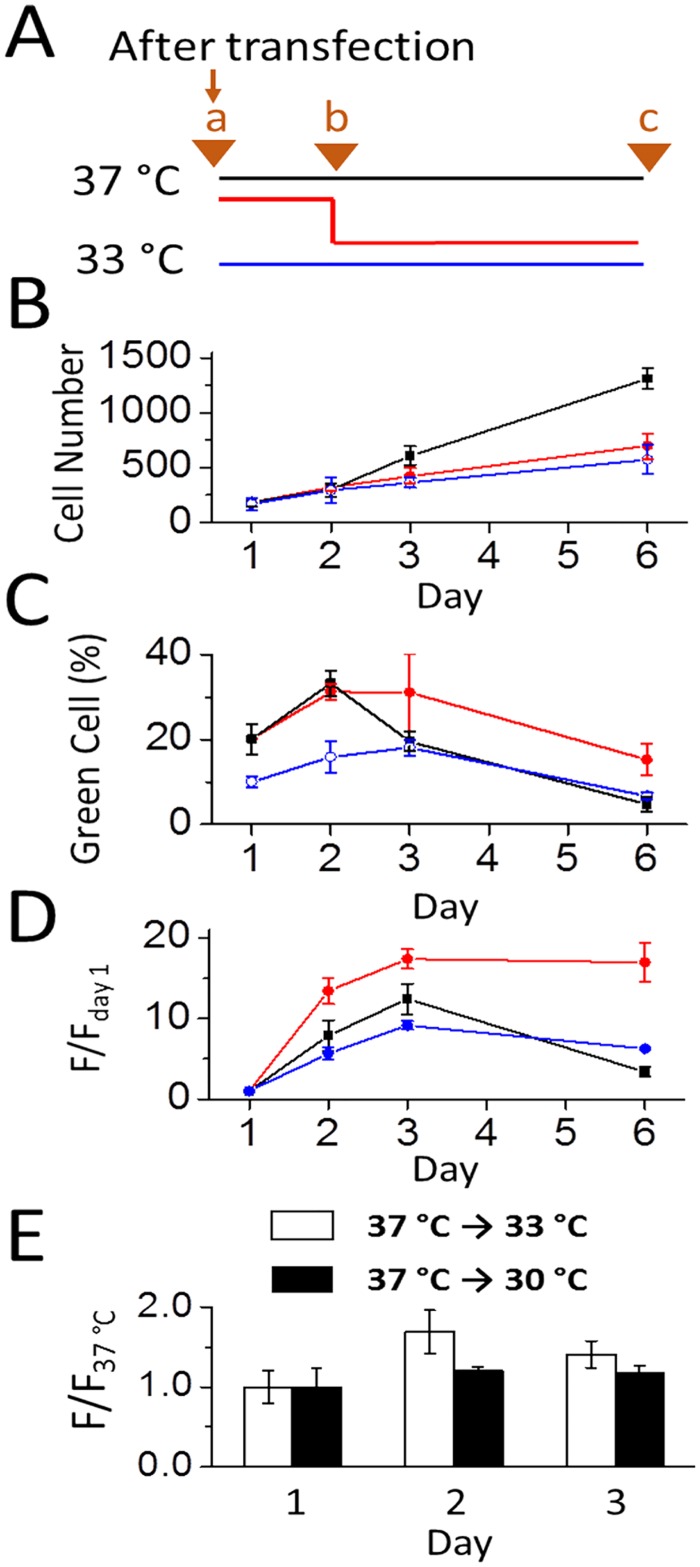
Time course of GFP expression in HEK-293S cells at 30°C, 33°C and 37°C. (A) Point ‘a’ represents the 5^th^ hour after transfection at which the medium was replaced (all the data were collected from cells transfected with 2 μg GFP plasmid/35 mm dish using calcium phosphate). Three sets of experiments began as reflected by three color-coded lines as in panels A-D, and in each set, triplicate dishes were used in our experiments. Specifically, blue line represents maintaining a culture at a 33°C incubator. Red line refers to the culture that first stayed at a 37°C incubator overnight (or 19 hours precisely) and then transferred to the 33°C incubator; this was labeled as point ‘b’ (note that point b correlates to day 1 in panels B-D). Black line represents the control (37°C culture). Point ‘c’ was the time (day 6) that the experiment ended. (B) The number of cells was counted from 34.8x bright-field images taken at days 1, 2, 3, and 6. Each data point was the average cell number counts of three 34.8x images taken at randomly selected locations of a dish. (C) The percentage of green cells in each experiment. Note that all data points were collected from the 34.8x images as in B. Both bright-field and fluorescence images were used for the determination of the transfection efficiency for each experiment. (D) The ratio of the green fluorescence intensity (F/F_day 1_). F_day 1_ is the fluorescence intensity for each culture at the 24^th^ hour. F stands for the fluorescence intensity at any particular time (day). All of the F/F_day 1_ values were collected from the fluorescence images as in (C). (E) The plot of the F/F_37°C_ values for days 1–3. All of the cells used for these experiments were transfected with GFP and maintained at 37°C for one day. Then, one set of the dishes were transferred to 33°C (hollow square), whereas another set dishes were transferred to 30°C (solid square). Therefore, this plot displaces the differential GFP expression on two different temperature scales, i.e., 33°C and 30°C, relative to 37°C.

We also followed the time course (days) of GFP expression and the enhancement of expression. Shown in Fig 5 as an example (where calcium phosphate transfection method was used), maintaining the culture at 33°C 24 hours after transfection, which is represented by the red line (Fig 5A), inhibited the growth rate (Fig 5B), as compared with the 37°C control culture (black line). Although the total cell count from the delayed (red line) and the immediate temperature shift to 33°C (blue line) after transfection was roughly similar at day 1 (Fig 5B), the delay temperature shift protocol resulted more green cells at the peak time (i.e., days 2 and 3) than the culture shifted to 33°C immediately after the medium replacement (red vs. blue line in Fig 5C). Those green cells in fact remained longer than those maintained at 37°C (red vs. black line in Fig 5C). Furthermore, at 33°C, green cells remained essentially undivided even at the end of the 6 day period, as compared with the 37°C culture (red line vs. black line in Fig 5D). Taken together, the delayed temperature shift protocol led to the enhancement of GFP expression with almost identical biomass on day 2 (Fig 5B and 5C, the black and red line at the 48th hour). Yet a mild hypothermic culture condition (i.e., 33°C) promoted a healthy temperature environment where a higher percentage of green cells survived longer (Fig 5C).

A further examination of the time course of green cell counts and fluorescence intensity (Fig 5) showed that a 19-hour delay of lowering culture temperature or the overnight recovery of the transfected culture at 37°C was essential in the use of this biphasic temperature culture protocol to enhance protein expression. This point becomes apparent when the time course of a delay temperature shift is compared with the immediate temperature drop from 37 to 33°C after the medium replacement (5 hours after transfection). As seen in Fig 5B, there was little difference in the total cell counts in both temperature change processes (red and blue lines) in the 6-day period. However, an immediate temperature drop led to the reduction of the transfection efficiency by roughly 50% (blue and red lines in Fig 5C). Furthermore, among the green cells expressed, the overall green fluorescence intensity did not even reach to the peak level of the control (blue line vs. black line for up to day 3 in Fig 5D). In fact, these results were consistent with the fluorescence images taken at 48 hours in Fig 2. Together, these results demonstrated that a 19-hour delay after medium replacement or a 24-hour delay after transfection before lowering the culture temperature from 37 to 33°C was critical for an increase in protein production. Immediate temperature drop after medium replacement or 5 hours after transfection adversely affected both transfection efficiency and protein production in HEK-293S cells. These results are consistent with the notion that growth arrest is unrelated to a higher protein expression at a sub-physiological culture temperature [23,24]. However, we do not yet understand the molecular mechanism of this delayed shift of culture temperature to 33°C in enhancing protein expression.

Because lowering culture temperature to 33°C led to a higher GFP expression, we asked whether dropping temperature even lower, i.e., to 30°C, would further increase GFP expression. Surprisingly, however, we found the opposite. Fig 5E is a comparison of the enhancement of GFP production in HEK-293S cells, represented by the ratio of the fluorescence intensity normalized to the control or 37°C culture. As seen, using the same mild hypothermic protocol, i.e., a 19-hr delayed shift to the respective temperature, green cells from a culture that was subject to 30°C essentially had a similar level of overall fluorescence intensity as the control. In contrast, at 33°C, HEK-293S cells produced more GFP than the control. These data suggested that lowering the culture temperature to 33°C, but not lower, led to a higher GFP expression. Furthermore, 30°C caused a dramatic reduction of cell counts, as we have described earlier (Fig 1).

When cells grew at 37°C, the number of green cells dropped more dramatically from day 2 to day 6 than cells growing either at 33°C or 30°C (Fig 5C, black line vs. either red or blue line). We note, however, that this reduction of green cell numbers was not due to cell death; in fact all cells continued to grow (Fig 5B), as the total cell counts continued to increase. Rather, the reduction of the green cells was due to that green cells began to divide as well, thereby “diluting” the percentage of green cells. Within the first three days, we did not observe any difference in cell viability. However, because we were interested in following the time course longer than 3 days for establishing the optimal condition, we replaced the cell culture media on day 4. From our data (Fig 5C and 5D), it is now clear, however, that within three days after transfection, the cells should be used, because the protein expression is peaked.

### Testing the mild hypothermic culture condition with PC12 cells

**Fig 6:**
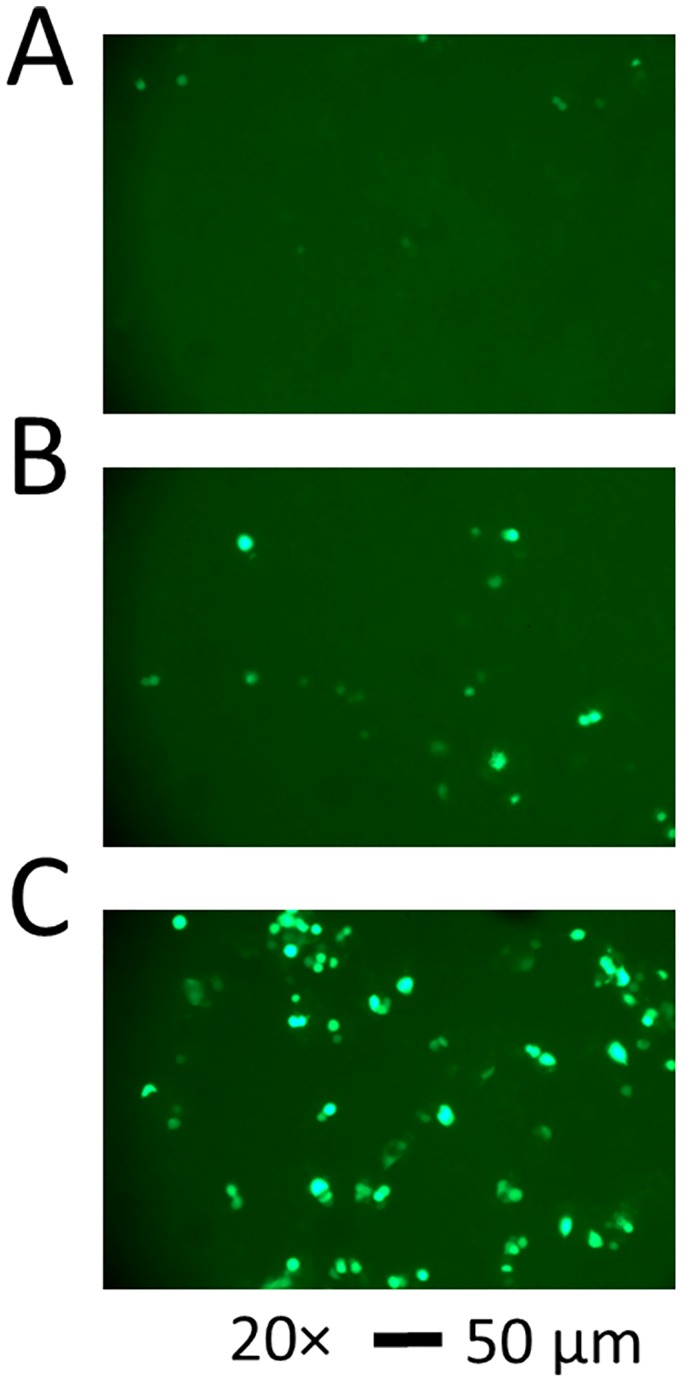
Fluorescence images of GFP-expressing PC12 cells. PC12 cells were transfected with 1 μg enhanced GFP plasmid DNA in 35 mm dishes using Lipofectamine 2000. Green fluorescence images were taken (34.8x magnification, 1/3 second exposure time, scale bars = 50 μm). In (A), the cells were maintained at 37°C, whereas in (B) the cells were transferred to 33°C on day 2. The images were taken at 96 hours after transfection. The percentage of the green cells was ~2% and ~6% for (A) and (B), respectively. In (C), when the cells were trypsinized during the passage or one day before transfection, the percentage of green cells was ~18%. As the comparison, the cells in (B) were not trypsinized. Like the cells in (B), the cells in (C) were also transferred to 33°C one day later.

To show the utility of the biphasic temperature culture protocol, which we established using HEK-293S cells, we further tested the same protocol with PC12 cells. PC12 cell line is derived from pheochromocytoma in the rat adrenal medulla and grows in culture as undifferentiated neuroblasts [39]. This cell line is a commonly used model system for studying neuronal development and function [40]. However, PC12 cells are known to be difficult to transfect by various transfection techniques, including chemical-based transfection methods [41]. Here we expressed GFP in PC12 cells, using Lipofectamine 2000, and one day later we shifted the culture to 33°C (Fig 6). By measuring the green fluorescence intensity, we first achieved ~6% transfection efficiency under the mild hypothermic culture condition (Fig 6B). In contrast, the transfection efficiency of the control culture, which was maintained at 37°C, was barely measurable (i.e., ~2%) (Fig 6A). When we used trypsin to treat the culture during the passage (or one day prior to transfection), which presumably helped dissociate cell clumps into single cells, the transfection efficiency reached ~18% under the mild hypothermic culture condition (Fig 6C) [41]. These results demonstrated that a delayed shift of culture temperature to 33°C could be useful for other types of cells and/or cells that are difficult to transfect and/or the yield of transfection efficiency is very low.

### Expression of AMPA receptors under the mild hypothermic culture condition and assay of the receptor property using whole-cell recording

We next tested if the same mild hypothermic culture condition could enhance membrane protein expression. Here we separately expressed two AMPA receptor subunits, GluA2 and GluA3 [27,42]. Transient expression of each subunit in HEK-293S cells leads to the formation of a functional, homomeric channel, and the channel property can be characterized using whole-cell recording [31,32] (note that the GluA2 channel we expressed was the unedited isoform in the Q/R editing site). Furthermore, we co-expressed GFP using a separate plasmid as a *cell marker* for whole-cell recording assay, because a green-colored cell would most likely express an AMPA receptor (the ratio of the plasmid used for GFP to an AMPA receptor was 1:5). The purpose of this experiment was to determine whether we could see an enhancement of AMPA receptors at a single-cell level.

**Fig 7:**
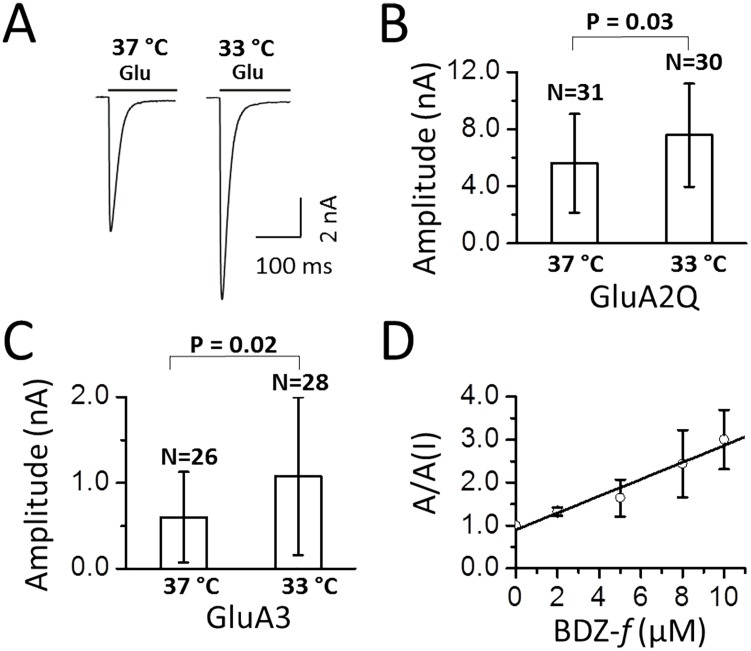
Whole-cell recording assay of AMPA receptors expressed in HEK-293S cells grown at 37°C and 33°C. All of the cells used for the assays were from 48 hours after transfection. (A) Representative traces of 3 mM glutamate induced whole-cell current from HEK-293S cells that transiently transfected with GluA2Q AMPA receptors. The cells cultured at 33°C were maintained at 37°C overnight first. (B) The average current amplitude from GluA2Q channels induced by 3 mM glutamate was shown for each culture. The number of the cells used for the recording was listed on top of each column. The error bar represents standard deviation from the mean. A two sample *t*-test on the whole-cell current data showed a significant difference between the cells cultured at 33°C and those cultured at 37°C (*P* value = 0.03). (C) The average whole-cell current amplitude from the GluA3 AMPA receptor channels induced by 3 mM glutamate. The number of cells used for the recording was listed on top of each column. Similarly, a two sample *t*-test also showed a significant difference between the data collected on the cells cultured at 33°C and those cultured at 37°C (*P* = 0.02). (D) The effect of BDZ-*f* on the GluA2Q AMPA receptor whole-cell current amplitude. The cells that expressed the channel were from the 33°C culture. The *K*_*I*_ value was found to be 5.1 ± 0.5 μM, determined from the slope (see detail in text).

Fig 7A is a pair of representative whole-cell current traces from two randomly chosen cells cultured on two different temperature scales. Based on the recording of ~30 cells from each culture for each receptor, we found that the whole-cell current amplitude increased by 1.4 ± 0.2 fold for GluA2Q (Fig 7B) and 1.8 ± 0.4 for GluA3 (Fig 7C) from the cells that were subject to the biphasic temperature culture, respectively, as compared to the control at 37°C for each receptor. Assuming the current amplitude was linearly proportional to the copy number of the receptors expressed on the cell surface, the range of the current amplitude increase suggested the HEK-293S cells produced on average ~1.6-fold more receptors when these cells were cultured at 33°C 24 hours after transfection than those cells maintained at 37°C. This value is consistent with what we found from the GFP experiment in Fig 3B: GFP expression level was ~1.4-fold higher from cells that were subject to the mild hypothermic treatment (the summary of these data is shown in Table 1). That AMPA receptor current amplitude measured at the single-cell level was higher from the cells growing at 33°C was also consistent with the conclusion from the GFP experiment, where the increase of green fluorescence intensity was attributed to the increase of GFP expression from “green” cells, rather than the increase of transfection efficiency. Therefore, ~1.4-fold higher GFP intensity reflected actually the increase of GFP expression at the single-cell (green cell) level.

**Table 1:** AMPA receptor properties.

	Amplitude (nA)		k_des_ (s^-1^)		t_rise_ (ms)	
	37°C	33°C	37°C	33°C	37°C	33°C
**GluA2** ^**a**^	5.6 ± 0.6	7.6 ± 0.7	108 ± 5	112 ± 5	1.2 ± 0.1	1.1 ± 0.1
**GluA3**	0.6 ± 0.1	1.1 ± 0.2	188 ± 7	192 ± 7	1.9 ± 0.1	1.6 ± 0.1

^a^For GluA2, the data were the average from 31 and 30 cells cultured at 37°C and 33°C, respectively. For GluA3, the data were the average from 26 and 28 cells cultured at 37°C and 33°C, respectively.

Also found in Table 1 is the comparison of the first-order rate constant of channel desensitization or *k*_des_ and the current rise time or the time it took for the whole-cell current to reach the peak amplitude (10–90%). No difference was observed in both parameters for the same receptor but from the cells grown normally at 37°C and those grown at 33°C for one day (or 48 hours after transfection). We also measured the inhibition constant for (-)-1-(4-aminophenyl)-4-methyl-7,8-methylenedioxy-4,5-dihydro-3-methylcarbamoyl-2,3-benzodiazepine or BDZ-*f* with GluA2Q [43]. BDZ-*f* is a known noncompetitive inhibitor of AMPA receptors [43]. The inhibition constant (*K*_*I*_) was found to be 5.1 ± 0.5 μM for the open-channel receptor conformation (Fig 7D), from the plot of the current amplitude in the absence and presence of an inhibitor or A/A(I) [43]. The *K*_*I*_ value was in good agreement with the inhibition constant of 5.4 ± 0.8 μM, which we reported before when we used the same receptor transiently expressed and cultured at 37°C [43]. All of these data (Fig 7 and Table 1) therefore illustrated that the HEK-293S cells expressed the same receptor with the same property regardless of whether these cells were grown at 37°C or the cells were shifted to a 33°C temperature environment 24 hours after transfection, except that the cells that were subject to 33°C treatment exhibited higher whole-cell current amplitude.

## Discussion

Animal cells and cell lines, such as HEK-293 cells, are commonly cultured at 37°C. However, as we have demonstrated in this study, lowering the culture temperature to 33°C 24 hours after transient transfection will give rise to ~1.5-fold higher expression of a recombinant protein, such as GFP and AMPA receptors. By following the time course of the GFP-expressing cells growing at 37°C normally and 33°C from 24 hours after transfection (including 19 hours recovery at 37°C in the normal growth medium), we found that a mild hypothermia (i.e., 33°C) increases cellular productivity of recombinant proteins while it reduces cell growth rate and suppresses cell division. As a result, more green cells remain undivided in a longer period of time. The property of a recombinant protein is unaffected, when the HEK-293S cells that express the protein are treated with a mild hypothermia, as shown by the use of two different AMPA receptors. Using PC 12 cells, we have further demonstrated that this method may be especially useful when a recombinant protein is difficult to express using a chemical-based transfection approach.

One important finding from our study is that the transfected culture after medium replacement must return to 37°C for overnight incubation or recovery (or 24 hours after transfection) before the culture temperature can be lowered to 33°C. Without this delayed temperature shift, both the transfection efficiency in HEK-293S cells and the total protein production (or the total intensity of GFP green fluorescence among all green cells) will be significantly reduced (Fig 5C and 5D), while the number of cells remains essentially the same (Fig 5B). While the molecular mechanism of this phenomenon is unclear, it appears that the initial translation of transiently transfected proteins is sensitive to temperature. Lowering it to 33°C too early or without an overnight incubation at 37°C will result in repression of cellular translation. Furthermore, even with an overnight delay but with the culture temperature lowered to 30°C, the cellular translation is similarly suppressed (Fig 5E). These data demonstrate that lowering temperature to 33°C, but not lower, can augment protein expression in HEK-293S cells, only when the transfected culture is allowed to “recover” at 37°C overnight. These phenomena are consistent with the hypothesis that growth arrest under mild hypothermic conditions (30–35°C) and the associated higher yield of protein expression in mammalian cells are two independent cellular responses [23,24].

As we have shown, HEK-293S cells that are subject to a delayed shift of culture temperature to 33°C one day after transfection produce ~1.5-fold higher expression of a recombinant protein. Such an enhancement is independent of transfection reagents, based on the test of calcium phosphate, Lipofectamine 2000, Lipofectamine LTX Plus, and Metafectene EASY. That said, Lipofectamine 2000 seems to be a better reagent when a lower amount of the cDNA is used for transfection (for GFP this amount can be as low as 0.5 μg/35 mm Petri dish). However, when a sufficient quantity of the DNA plasmid is used (e.g., 5 μg GFP/35 mm Petri dish), the calcium phosphate method is as efficient as Lipofectamine 2000. Because lower temperature does not affect the transfection efficiency, but reduces significantly the rate of cell growth, the increase in protein expression should be attributed to the increase of protein expression at the single cell level. This is consistent with our observation in that (a) green cells become greener when GFP is expressed in HEK-293S cell, and (b) cells expressing AMPA receptors at lower temperature exhibit higher whole-cell current amplitude. The phenomenon can be best explained by the notion that low temperature induces cell growth suppression due to cell cycle arrest at the G(0)/G(1) phase [17,18,21,22]. In fact, cell cycle arrest, particularly in G1 phase, typically using a low temperature culture, has been a well-practiced means to enhance protein yield in production cultures, such as in CHO cells [6].

The method we have described should be simple to implement. For static culture or a small-scale culture, two incubators with two culture temperatures (i.e., one is at 37°C and the other is at 33°C) may be the best way. We have shown previously that using HEK-293S cells in static culture is suitable for electrophysiological measurements, where only the dish-attached cells are selected to make gigaohm seals (in static culture, the majority of 293S cells are attached to Petri dishes, albeit more loosely than either regular 293 or 293T cells) [33]. Furthermore, that the mild hypothermic treatment of a static culture transfected with AMPA receptors can enhance receptor expression on a single cell level by up to ~1.5-fold is also consistent with the level of GFP increase when green fluorescence is used as a “reporter signal”. Therefore, it appears that between 37°C and 33°C, the biosynthesis of AMPA receptors, their processing, surface delivery and turnover are not affected or they are not affected differentially. It is known, for example, the endocytosis can be affected by lowering temperature, but temperature will have to be lowered to a single digit [44]. Therefore, our method should be applicable for other membrane proteins, such as other channel proteins. Although we did not test the suspension culture using HEK-293S cells, this cell line is suitable for suspension culture in shake flasks and/or bioreactors for a large-scale production of proteins per unit culture volume [35,45,46]. In suspension culture, changing temperature without changing culture vessels is feasible, because the response of cells to temperature is independent of culture size. Finally, the use of 293S cells may be especially advantageous for preparing membrane proteins, because this cell line is known to express proteins whose activities are modulated by complex post-translation, a feature often observed for membrane proteins [47].
